# Comparative Efficacy of Acupuncture-Related Techniques for Urinary Retention After a Spinal Cord Injury: A Bayesian Network Meta-Analysis

**DOI:** 10.3389/fneur.2021.723424

**Published:** 2022-02-07

**Authors:** Kelin He, Xinyun Li, Bei Qiu, Linzhen Jin, Ruijie Ma

**Affiliations:** ^1^Key Laboratory of Acupuncture and Neurology of Zhejiang Province, Third School of Clinical Medicine (School of Rehabilitation Medicine), Zhejiang Chinese Medical University, Hangzhou, China; ^2^Department of Acupuncture and Moxibustion, The Third Affiliated Hospital of Zhejiang Chinese Medical University (Zhongshan Hospital of Zhejiang Province), Hangzhou, China

**Keywords:** acupuncture, urinary retention, network meta-analysis, spinal cord injury, clinical efficacy

## Abstract

**Background:**

Urinary retention is one of the most frequent complications of spinal cord injuries (SCI) and negatively impacts patient satisfaction and quality of life. Acupuncture as an integral part of traditional Chinese medicine (TCM) has recently drawn widespread attention for its potential in the management of urinary retention. However, there are many different styles of acupuncture-related techniques, and the optimal choice of acupuncture for urinary retention after SCI is still unclear. Hence, this study uses a Bayesian network meta-analysis (NMA) to compare the efficacy of different types of acupuncture therapies using both direct and indirect evidence.

**Methods:**

Randomized controlled trials of acupuncture-related techniques for treating urinary retention after SCI were retrieved from the following electronic databases: Pubmed, Cochrane Library, Web of Science, China National Knowledge Infrastructure (CNKI), the Chinese Biomedical Literature Service System (SinoMed), the Wan-Fang database, and the Chinese Scientific Journals Database (VIP). The retrieval time was from inception to November 2020. Clinical effective rate (CER) was the primary outcome indicator and residual urine volume (RUV) was the secondary outcome indicator. A Bayesian NMA was performed using the Markov chain Monte Carlo method in R software (version 3.6.1) interfacing with JAGS software (version 4.3.0). The node-splitting method was used to identify inconsistencies. In addition, a comparative adjusted funnel plot was used to assess publication bias.

**Results:**

A total of 26 randomized controlled trials involving 1,652 patients were included. Bayesian NMA showed that electroacupuncture combined with moxibustion ranks first in both CER and RUV. In addition, in terms of cumulative probability, electro-acupuncture combined with moxibustion ranked first in CER. The results of the node splitting method revealed that direct and indirect evidence were consistent (*P* > 0.05). In addition, publication bias was detected.

**Conclusion:**

A Bayesian NMA that combined direct and indirect comparisons showed that electro-acupuncture combined with moxibustion had a better effect on urinary retention due to SCI. However, it still needs a large sample size and high-quality randomized controlled trials to verify this finding.

**Systematic Review Registration:** https://inplasy.com/, identifier: INPLASY2021110005.

## Introduction

Urinary retention is impaired voiding despite a full bladder, leading to a post-void residual (PVR) (1). It is one of the most frequent results of spinal cord injury (SCI) and negatively impacts patient satisfaction and quality of life. Urinary retention after SCI refers to dysfunction of the urinary bladder due to damaged bladder neural circuits following SCI. Studies have found that the bladder wall appears ischemic following SCI, affecting bladder metabolic function and resulting in the inability to discharge urine (2). Urinary retention has been closely associated with adverse outcomes including urinary tract infections, overdistension of the urinary bladder, and high mortality rates (3–5). Urethral catheterization and bladder function training are currently the main treatments for urinary retention among those SCI patients whose normal bladder function is altered. However, urethral catheterization is strongly associated with urinary tract infection (UTI), and the risk of a UTI increases with how long the patient catheterizes (6). Catheter-associated UTIs are the most common nosocomial infections. Catheter-associated UTIs affect men and women, and long-term urinary catheterization always and inevitably leads to bacteria in the urine of both sexes. Long-term catheterization typically results in a daily risk of 3–7% for the development of symptomatic catheter-associated UTI (7). In contrast, bladder function training leads to limited functional improvement. There is therefore a strong demand for novel and effective therapies for urinary retention after SCI.

Acupuncture as an integral part of traditional Chinese medicine (TCM) has recently drawn widespread attention for its potential in the management of urinary retention. It has consequently been the subject of research works on the topic (8–12). Advantages of acupuncture, as non-pharmacological therapy, including safety, convenience, and minimal side effect profile (13, 14). In China, many domains of acupuncture such as manual acupuncture, electro-acupuncture, moxibustion therapy, auricular acupuncture, and acupoint patching are widely used in the treatment of urinary retention after SCI. A previous traditional pairwise meta-analysis indicated that acupuncture has a positive effect on urinary retention due to SCI (15). However, there are many different styles of acupuncture, and the optimal acupuncture intervention is still unclear. Network meta-analysis (NMA) based on the traditional pairwise meta-analysis is an increasingly popular tool that can simultaneously synthesize direct and indirect evidence by summarizing different interventions for the same disease (16, 17). NMA can also assess the efficacy of different treatments and estimate the relative efficacy of such interventions (18, 19). Therefore, this study aimed to use NMA to explore the efficacy of different acupuncture therapy types in the treatment of urinary retention after SCI. This work may help provide guidelines for acupuncture therapy in the treatment of urinary retention after SCI and serve as the basis of future work.

## Materials and Methods

This study followed the Preferred Reporting Items for Systematic Reviews and Meta-Analyses (PRISMA) guidelines (20), and the study protocol has been registered on the website of https://inplasy.com/ (Registration number: INPLASY2021110005).

### Eligibility and Exclusion Criteria

**Literature inclusion:** The PICOS framework (participants, interventions, comparisons, outcome, and study design) was used to identify literature appropriate for inclusion in this work. Eligible literature were randomized controlled trials (RCTs): (1) Study design: only articles referring to RCTs were included; (2) Participants: diagnosed with spinal cord injury, survived the shock period, not limited by age, gender, race, or nationality; (3) Intervention and control measures: using a similar study as the reference (21), acupuncture-related techniques were defined as acupoint-based therapy (e.g., manual acupuncture, electro-acupuncture, auricular acupuncture, moxibustion, acupoint patching, acupoint injection, acupoint embedding, and warm needling moxibustion) in this systematic review, regardless of stimulation method. The control group received conventional therapy or conventional therapy combined with some other therapy. The current conventional-therapies strategy for urinary retention after SCI consists of intermittent catheterization and bladder function training. (4) Outcome indicators: The primary outcome was Clinical effective rate (CER). Based on the presence of clinical symptoms and objective indicators, efficacy was divided into valid and invalid categories. No improvement in clinical symptoms, including those that worsen, was considered invalid. CER = (total number – invalid number)/total number × 100% (22). The secondary outcome was residual urine volume (RUV). The level of urinary retention was evaluated by the amount of RUV.

**Literature exclusion:** (1) Treatment measures in the experimental group included non-acupuncture-related therapies such as Chinese medicine and Western medicine; (2) The trial data was wrong; (3) The trial data was repeated; (4) The full text could not be obtained; (5) The outcome was not relevant.

### Search Strategy

RCTs of acupuncture-related techniques for the treatment of urinary retention after SCI were extracted from the following electronic databases: China National Knowledge Internet (CNKI), Wan-fang Database, Chinese Scientific Journals Database (VIP), the Chinese Biomedical Literature Service System (SinoMed), PubMed, Cochrane Library, and Web of Science. The retrieval period was from inception to November 2020. Terms such as “acupuncture,” “manual acupuncture,” “electro-acupuncture,” “scalp needle,” “elongated needle,” “moxibustion,” “warm needling,” “acupuncture plus moxibustion,” “acupoint injection,” “acupoint patching,” “auricular acupuncture,” “ear acupuncture,” “spinal cord injury,” “urinary retention,” and “neurogenic bladder” were used as subject words, keywords, free-text terms, or MeSH (Medical Subject Heading) terms to identify potentially eligible studies. The search strategy was adjusted for each database. There were no restrictions on blinding methods, language, and year of publication.

### Data Extraction and Quality Assessment

Relevant data from the eligible studies were extracted by two independent reviewers, and Microsoft Excel 2019 (Microsoft Corp, Redmond, WA, USA) was used to manage the data. A standard form table was constructed that included publication information (authors, publish date), demographic data (gender, age, ASIA grading, sample size, the course of SCI onset), intervention measures (experimental group: acupuncture treatments plus conventional therapy; control group: conventional therapy or conventional therapy plus other acupuncture therapy), and outcome (CER, RUV). The independent reviewers assessed the quality of the included trials using the Cochrane risk of bias tool (23). The Cochrane Risk of Bias tool includes seven items: (1) random sequence generation; (2) allocation concealment; (3) blinding of participants and personnel; (4) blinding of outcome assessment; (5) incomplete outcome data; (6) selective reporting; (7) other sources of bias. Each trail was graded as either “low,” “high,” or “unclear” risk. During trial selection when data extraction and quality assessment scores were inconsistent, discrepancies were resolved by a third reviewer.

### Statistical Analysis

Given the potential sources of clinical heterogeneity among the included studies, a random effect model was adopted to merge the datasets. The Bayesian meta-analysis was performed using R software (version 3.6.3; http://www.Rproject.org) and JAGS software (version 4.3.0, https://nchc.dl.sourceforge.net/project/mcmc-jags/JAGS/4.x/Windows/JAGS-4.3.0.exe), using the Bayesian hierarchical model and the Markov Chain Monte Carlo algorithm (24). We used 200,000 iterations, and the first 5,000 iterations were regarded as burn-in for annealing to eliminate the influence of the initial value. The combined results were presented as odds ratios (ORs) with 95% confidence intervals (95% CIs) for dichotomous outcomes. Due to the limitations of dichotomous outcomes, the description of “healing,” “remarkable effect,” and “effective” described in the study were combined into valid. The combined results were presented as mean differences (MDs) with 95% CIs for continuous outcomes. If 95% CIs of ORs did not contain 1 and 95% CIs of MDs did not contain 0, the corresponding ORs or MDs were considered to indicate a statistically significant difference. The surface under the cumulative ranking area (SUCRA) was used to rank the probabilities for different interventions. The SUCRA values range from 0 to 100%, assigned to the worst and best treatments (25), respectively. Publication bias and small-study effects among the included RCTs for the primary outcome were compared using an adjusted funnel plot (26).

## Results

### Literature Selection

**Figure 1:**
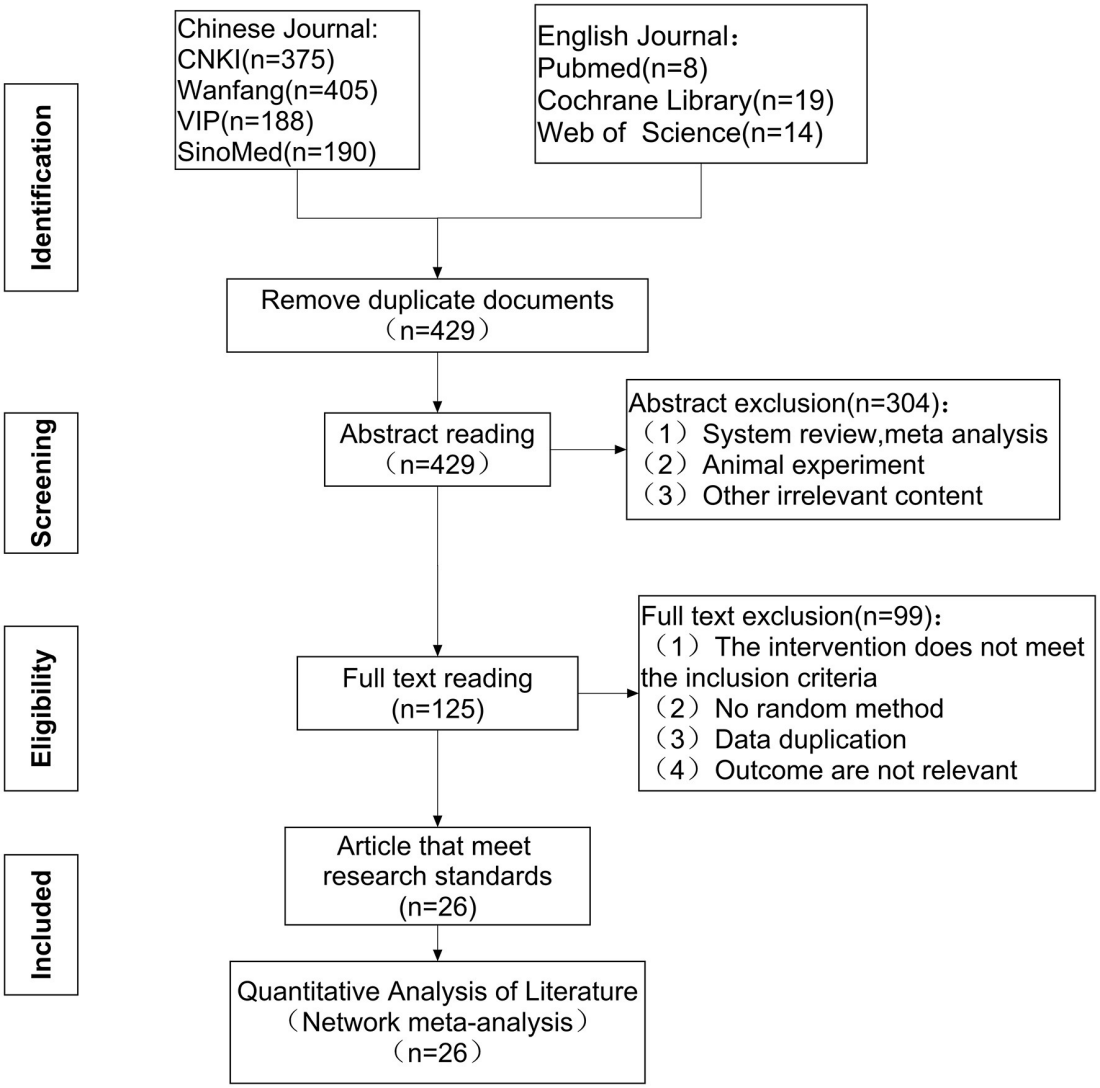
Flow diagram depicting the selection process of eligible studies. CNKI, China national knowledge infrastructure; VIP, Chinese Scientific Journals Database; SinoMed, Chinese Biomedical Literature Service System; n, number of publications.

A total of 1,199 references were identified (375 references from CNKI, 405 references from Wanfang, 188 references from VIP, 190 references from SinoMed, 8 references from Pubmed, 19 references from Cochrane Library, and 14 references from Web of Science) and imported into Endnote X9 (Clarivate Analytics, Philadelphia, PA, USA). After eliminating duplicates, 419 articles remained. Following the exclusion of reviews, case reports, animal experiments, and other irrelevant content, 125 studies remained. Non-randomized methodologies, data duplication, mixed interventions, and outcome indicators that did not include CER or RUV were also excluded. A total of 26 RCTs were ultimately included after evaluating the full text. A detailed flowchart depicting the article screening process is shown in Figure 1.

### Study Characteristics

A total of 26 articles were included, of which 25 trials (27–51) were double-arm RCTs and one trial (11) was three-arm RCTs. The total sample consisted of 1,652 patients (805 in the control group and 847 in the treatment group). Eight studies did not mention American Spinal Injury Association (ASIA) grade. Six studies did not report the course of SCI. Three studies did not mention the gender ratio of the participants. Two studies only reported the overall gender ratio. Three studies did not report patient age. The interventions in the control group included conventional therapy (CT) combined with electro-acupuncture (EA), CT combined with drug, CT combined with warm needle moxibustion (WNM), and CT combined with manual acupuncture (MA). The interventions in the experimental group included CT combined with EA, CT combined with Moxibustion (MOX), CT combined with MA, CT combined with acupoint patching (AP), CT combined with MOX, and Dong Shi Qi Point (DSQP), CT combined with auricular acupuncture (AA), CT combined with EA and MOX, CT combined with MOX and Wrist-ankle acupuncture (WAA). The shortest treatment course was 7 days and the longest was 2 months. Twenty-one trials reported CER and 20 trials reported RUV. Detailed study summaries are shown in Table 1.

**Table 1:** Characteristics of the included studies.

**References**	**Sample size**	**ASIA grade**				**Intervention protocol**		**Outcomes**
			**The course of SCI onset**	**Gender(M/F)**	**Average age** **(years)**	**Control group**	**Experimental group**	
Zhu et al. (27)	C:20; E:20	–	C:(4.92 ± 2.46) m; E:(5.60 ± 3.12) m	C:14/6; E:13/7	C:44.23 ± 11.67; E:45.85 ± 12.16	CT (not very clear) +EA (EA at bilateral Huiyang (BL35) and Ciliao (BL32); treatment duration was 30 min, 6 times per week for 4 weeks)	CT +EA (consistent with the control group) +MOX (heat-sensitive MOX at the sacral and caudal regions, 6 times per week for 4 weeks)	CER, RUV
Zhao (28)	C:20; E:20	BCD	C:(3.72 ± 1.44) m; E:(3.21 ± 1.35) m	C:15/5; E:16/4	C:40 ± 8; E:35 ± 9	CT (routine rehabilitation) +EA (EA at Jiaji (EX-B2); treatment duration was 30 min, once a day for 50 consecutive days)	CT +EA (EA at Jiaji (EX-B2)) +MOX (needle warming MOX at Jiaji (EX-B2))	CER, RUV
Zhang et al. (29)	C:34; E:34	BCD	C:(2.8 ± 1.1) m; E:(2.7 ± 1.3) m	C:28/6; E:26/8	C:39 ± 6; E:39 ± 5	CT (intermittent catheterization and bladder training)	CT +MOX (herb-partitioned MOX at Shenque (CV8), Guanyuan (CV4), Qihai (CV6), and Zhongji (CV3); treatment duration was 30 min, 7 times per week for 8 weeks)	CER, RUV
Wang et al. (32)	C:30; E:30	BCD	C:(28.1 ± 15.6) d; E:(26.5 ± 14.9) d	C:18/12; E:16/14	C:42.3 ± 6.7; E:43.3 ± 5.7	CT (intermittent catheterization and bladder training) + MA (MA at Pangguangshu (BL28), Guanyuan (CV4), and Zhongji (CV3); treatment duration was 20 min, once a day for 20 consecutive days)	CT (consistent with the control group) +MOX (heat-sensitive MOX at Pangguangshu (BL28), Guanyuan (CV4), Zhongji (CV3), once a day for 20 consecutive days)	CER
Tong et al. (34)	C:30; E:28	BC	C:(44.03 ± 8.33) d; E:(43.34 ± 9.12) d	C:19/11; E:18/12	C:36.20 ± 5.09: E:32.5 ± 8.6	CT (intermittent catheterization and bladder training)	CT +EA (EA at Zhongji (CV3), Guanyuan (CV4), and Sanyinjiao (SP6); treatment duration was 30 min, 6 times per week for 8 weeks)	RUV
Sheng et al. (37)	C:30; E:30	BCD	C:(2.8 ± 1.1) m; E:(2.6 ± 1.2) m	C:18/12; E:16/14	C:45.2 ± 3.5; E:45.3 ± 1.4	CT (intermittent catheterization and bladder training)	CT +MOX (herb-partitioned MOX at Shenque (CV8), Guanyuan (CV4), treatment duration was 30 min, 7 times per week for 8 weeks)	RUV
Li et al. (41)	C:35; E:35	BCD	–	C:20/15; E:22/13	C:33.8 ± 2.6; E:36.8 ± 1.5	CT (intermittent catheterization and bladder training)	CT +WAA (bilateral Ankole region) +MOX (ginger-separated MOX at Zhongji (CV3), once a day for 20 consecutive days)	CER, RUV
Gao and Cai (50)	C:30; E:30	BCD	–	C:22/8; E:18/12	C:36.8 ± 2.9; E:35.8 ± 1.3	CT (intermittent catheterization and bladder training)	CT +MOX (gentle MOX at Zhongji (CV3), Guanyuan (CV4), Shuifen (CV9))	CER, RUV
Yang et al. (30)	C:20; E:20	ABCD	C:(3.0 ± 1.1) m; E:(3.1 ± 1.3) m	C:16/4; E:17/3	C:29.1 ± 5.6; E:28.9 ± 5.1	CT (intermittent catheterization and bladder training)	CT +MOX (herb-partitioned MOX at Shenque (CV8), twice a week for 8 weeks)	CER, RUV
Wu et al. (31)	C:64; E:68	ABCD	C:(14.3 ± 4.4) m; E:(13.6 ± 3.9) m	C:39/25; E:41/27	C:31.4 ± 8.5; E:29.7 ± 17. 4	CT (intermittent catheterization)	CT+EA (EA at Qihai (CV6), Guanyuan (CV4), Zhongji (CV3), Qugu (CV2), Sanyinjiao (SP6), Baliao (BL31-34), Yinlingquan (SP9), Pangguangshu (BL28), and Shenshu (BL23); treatment duration was 20 min, 6 times per week for 2 weeks)	CER
Wang et al. (33)	C:18; E:18	–	C:(12~41) d; E:(11~41) d	C:16/2; E:15/3	C:48.6 ± 10.0; E:47.5 ± 10.2	CT (intermittent catheterization and bladder training)	CT+multiple acupuncture (Morning: MA at Guanyuan (CV4) Afternoon: EA at Zhongliao (BL33); treatment duration was 30 min, once a day for 10 consecutive days)	CER
Su (35)	C:34; E:32	BCD	C: ≤ 4 w; E: ≤ 4 w	–	–	CT (intermittent catheterization)	CT+MA (Zhibian (BL54) toward the direction of Shuidao (ST28); treatment duration was 30 min, once every other day for 30 days)	CER, RUV
Su (36)	C:31; E:31	ABCD	C:(35.67 ± 8.29) d; E:(36.12 ± 4.83) d	C:26/5; E:24/7	C:41.87 ± 13.88; E:40.25 ± 14.06	CT (not very clear) +Drug (3 mg neostigmine, once a day for 6 consecutive days)	CT+EA (EA at Baliao (BL31-34); treatment duration was 30–40 min, 6 times per week for 2–3weeks)	CER, RUV
Ma (38)	C:31; E:30	AB	C:(67.61 ± 17.04) d; E:(66.20 ± 13.14) d	C:21/10; E:22/8	C:34.48 ± 9.04; E:34.23 ± 9.62	CT (intermittent catheterization)	CT +MOX (herb-partitioned MOX at Shenque (CV8), Guanyuan (CV4), Zhongji (CV3), Zusanli (ST36), Mingmen (GV 4), Shenshu (BL23), and Pangguangshu (BL28); treatment duration was 20 min, 6 times per week for 5 weeks)	CER, RUV
Luo (39)	C:30; E:30	ABCD	C:(3.12 ± 0.81) m; E:(3.56 ± 0.79) m	C:13/17; E:16/14	C:40.25 ± 5.12; E:39.87 ± 5.61	CT (intermittent catheterization and bladder training)	CT +MA (MA at Zhongji (CV3), Qihai (CV6), Guanyuan (CV4), Guilai (ST29), Shuidao (ST28), Yinlingquan (SP9), and Sanyinjiao (SP6); treatment duration was 30 min, once a day for 28 days)	CER, RUV
Li et al. (40)	C:30; E:30	–	–	–	–	CT (catheterization)	CT +multiple acupuncture (Morning: MA at Guanyuan (CV4), Afternoon: EA at Ciliao (BL32), and Zhongliao (BL33); treatment duration was 30 min, once a day for 10 days)	CER
Kuang et al. (42)	C:30; E:30	–	–	C:21/9; E:19/11	C:44.7 ± 3.9; E:45.1 ± 3.7	CT (intermittent catheterization and bladder training)	CT+MOX (box MOX at Shenque (CV8), Guanyuan (CV4), Qihai (CV6), Zhongji (CV3), Shenshu (BL23), Weizhong (BL40), Sanyinjiao (SP6), and Zusanli (ST36); treatment duration was 30 min, once a day for 30 days)	CER, RUV
Jiang et al. (43)	C:25; E:26	–	(7.42 ± 5.13) m	28/23	36.20 ± 8.64	CT (not very clear) +WNM (WNM at Qihai (CV6), Guanyuan (CV4), and Ciliao (BL32), the treatment duration was 40 min, once a day for 60 days)	CT+MOX (heat-sensitive MOX at Qihai (CV6), Guanyuan (CV4), and Ciliao (BL32); treatment duration was 40 min, once a day for 60 days)	CER
Huo et al. (44)	C:30; E:30	–	–	C:25/5; E:26/4	C:34.62 ± 1.85; E:36.78 ± 2.32	CT (routine rehabilitation)	CT +AP (AP at Qihai (CV6), Guanyuan (CV4), Zhongji (CV3), Yinlingquan (SP9), Sanyinjiao (SP6), Shenshu (BL23), and Pangguangshu (BL28), once a day for 7 consecutive days)	CER, RUV
Hu and Wang (45)	C:44; E:45	–	(1–4) m	C:23/21; E:25/20	C:36.1; E:35.3	CT (intermittent catheterization) +Drug (0.5–1 mg neostigmine, once a day for 7 consecutive days)	CT+MA (MA at Shenshu (BL23), Ciliao (BL32), Yinlingquan (SP9), Sanyinjiao (SP6), and Zhongji (CV3); treatment duration was 30 min, once a day for 7 consecutive days)	CER
Hou et al. (46)	C:32; E:32	–	C:(8.84 ± 2.94) d; E:(8.31 ± 2. 51) d	C:22/10; E:25/7	C:41 ± 9.36; E:40.53 ± 10.76	CT (intermittent catheterization)	CT +DSQP (the treatment duration was 30 min, once a day for 7 consecutive days) +MOX (herb-partitioned MOX at Shenque (CV8), once a day for 30 days)	CER, RUV
Guo (47)	C:30; E:30	A	–	46/14	–	CT (intermittent catheterization)	CT+EA (EA at Shuidao (ST28), Yinlingquan (SP9), Ciliao (BL32), and Pangguangshu (BL28); treatment duration was 30 min, 6 times per week for 6 weeks)	CER, RUV
Gao et al. (48)	C:30; E:32	BCD	C:(46.03 ± 8.33) d E:(48.34 ± 10.12) d	C:16/14; E:15/17	C:35.20 ± 8.12; E:37.20 ± 7.09	CT (voiding and bladder training)	CT+MA (MA at Qihai (CV6), Guanyuan (CV4), Zhongji (CV3), Yaoyangguan (GV3), and Mingmen (GV 4); treatment duration was 30 min, 6 times per week for 8 weeks)	RUV
Gao et al. (49)	C:30; E:30	BCD	C:(33.57 ± 17.89) d; E:(35.17 ± 15.48) d	C:17/13; E:14/16	C:45.2 ± 11.43; E:43.7 ± 10.89	CT (voiding and bladder training)	CT+EA (EA at Qihai (CV6), Guanyuan (CV4), Zhongji (CV3), Yaoyangguan (GV3), and Mingmen (GV 4); treatment duration was 30 min, 6 times per week for 4 weeks)	RUV
Bu et al. (51)	C:32; E:34	ABCD	C:(10 ± 4.8) d; E:(11 ± 3.9) d	C:28/4; E:29/5	C:37.4 ± 16.3; E:38.2 ±15.1	CT (intermittent catheterization)	CT+AA (AA at bladder, ureter, kidney, cervical spine, thoracic spine, and lumbosacral spine point; treatment duration was 30–60 min, 20 times for 25 days)	CER, RUV
Gu et al. (11)	C:35; EA:34; SA:38	BCD	C:(22.2 ± 2.4) d EA:(25.8 ± 2.4) d SA:(25.5 ± 2.5) d	–	C:40.6 ± 9.8; EA:39.6 ± 7.6 SA:40.75 ± 12.5	CT (behavioral interventions, such as fluid schedules and regular voiding attempts, clean intermittent catheterization)	CT+EA (EA at Shangliao (BL31), Xialiao (BL34); treatment duration was 20 min) SA (the needle was taped to the dermal surface of Baliao (BL31-34) using an adhesive tape without insertion, serving as a mock EA therapeutic instrument)	RUV

*C, control group; E, experimental group; m, month; d, day; w, week; CT, conventional therapy; EA, electro-acupuncture; MOX, moxibustion; WNM, warm needle moxibustion; AA, auricular acupuncture; AP, acupoint patching; DSQP, Dong Shi Qi point; MA: manual acupuncture; WAA, wrist-ankle acupuncture; SA, sham acupuncture; RUV, residual urine volume; CER, clinical effective rate*.

### Quality Evaluation

**Figure 2:**
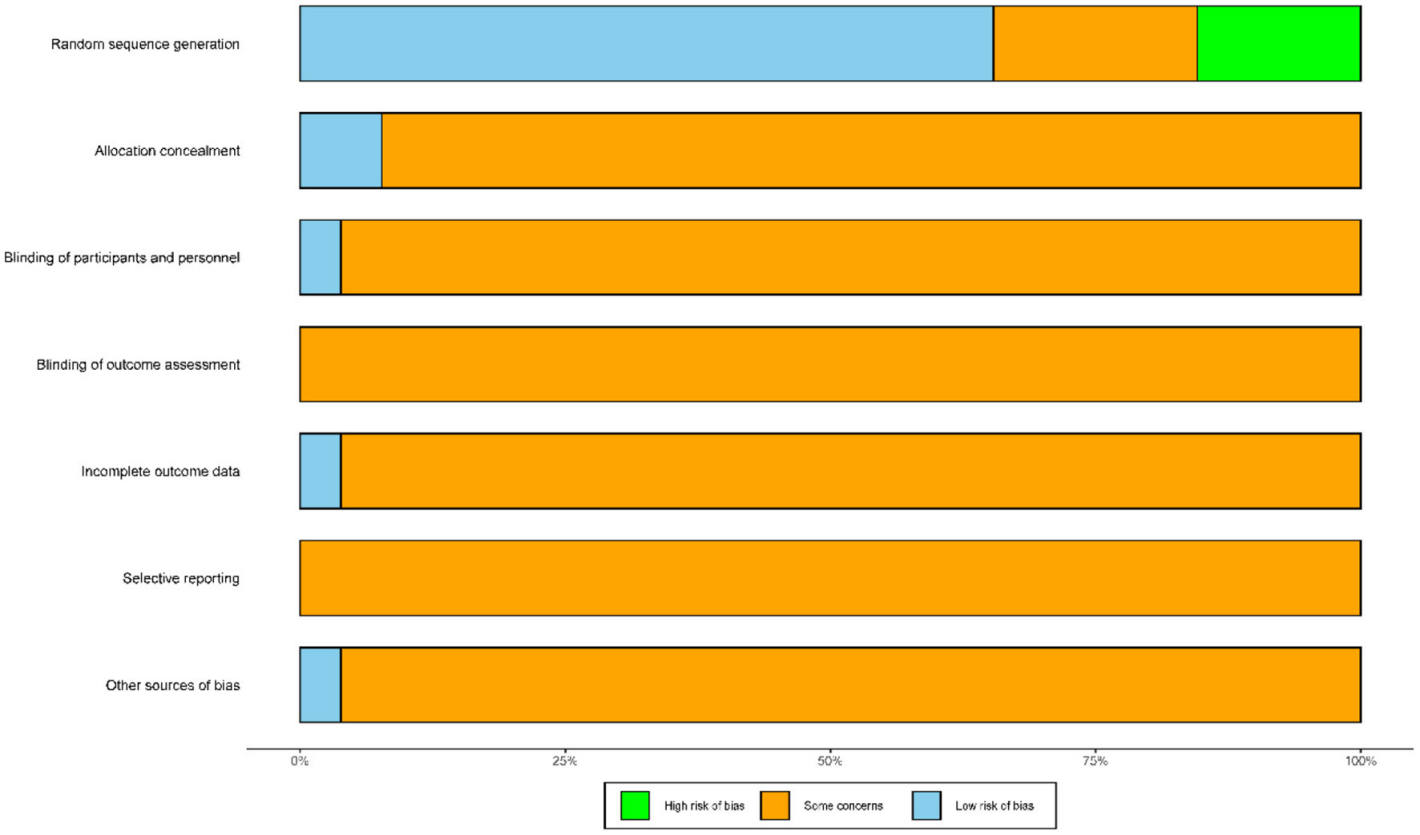
Risk of bias of the included studies. The vertical axis represents the quality evaluation items and the horizontal axis represents the number of studies.

Figure 2 depicts the risk of bias. For random sequence generation, 13 trials used random number tables, three trials used network programming software, six trials did not provide randomization details, and four trials used a wrong random method. Two trials involved allocation concealment and were assigned a low risk of bias. Only one trial mentioned single blindness and was assigned a low risk of bias. Only one trial reported two cases dropped out and the influence of incomplete outcome data was assigned a low risk of bias. All trials that did not mention study protocol and the influence of selective reporting were assigned an “uncertain” risk of bias. Only one trial reported disclosure of conflict of interest and the influence of other sources of bias was assigned a low risk of bias.

### Outcome

#### Clinical Effectiveness

**Figure 3:**
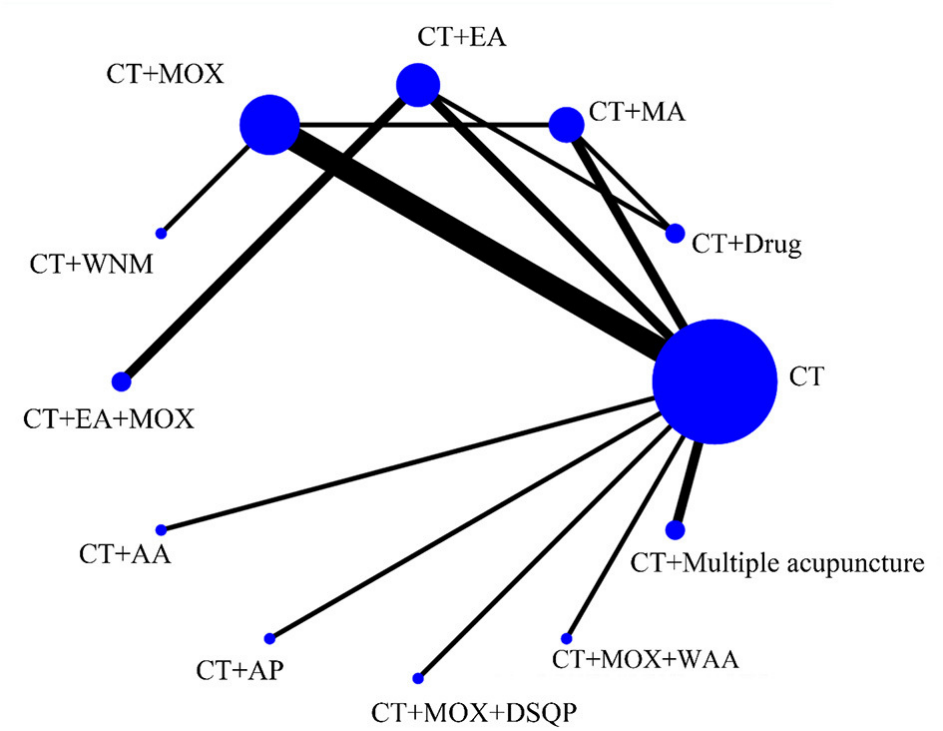
Network of eligible comparisons for the network meta-analysis of CER. Each node represents an intervention and the size of each node represents the number of randomly assigned participants. Each line represents a direct comparison between interventions and the width of the lines represents the number of studies.

**Figure 4:**
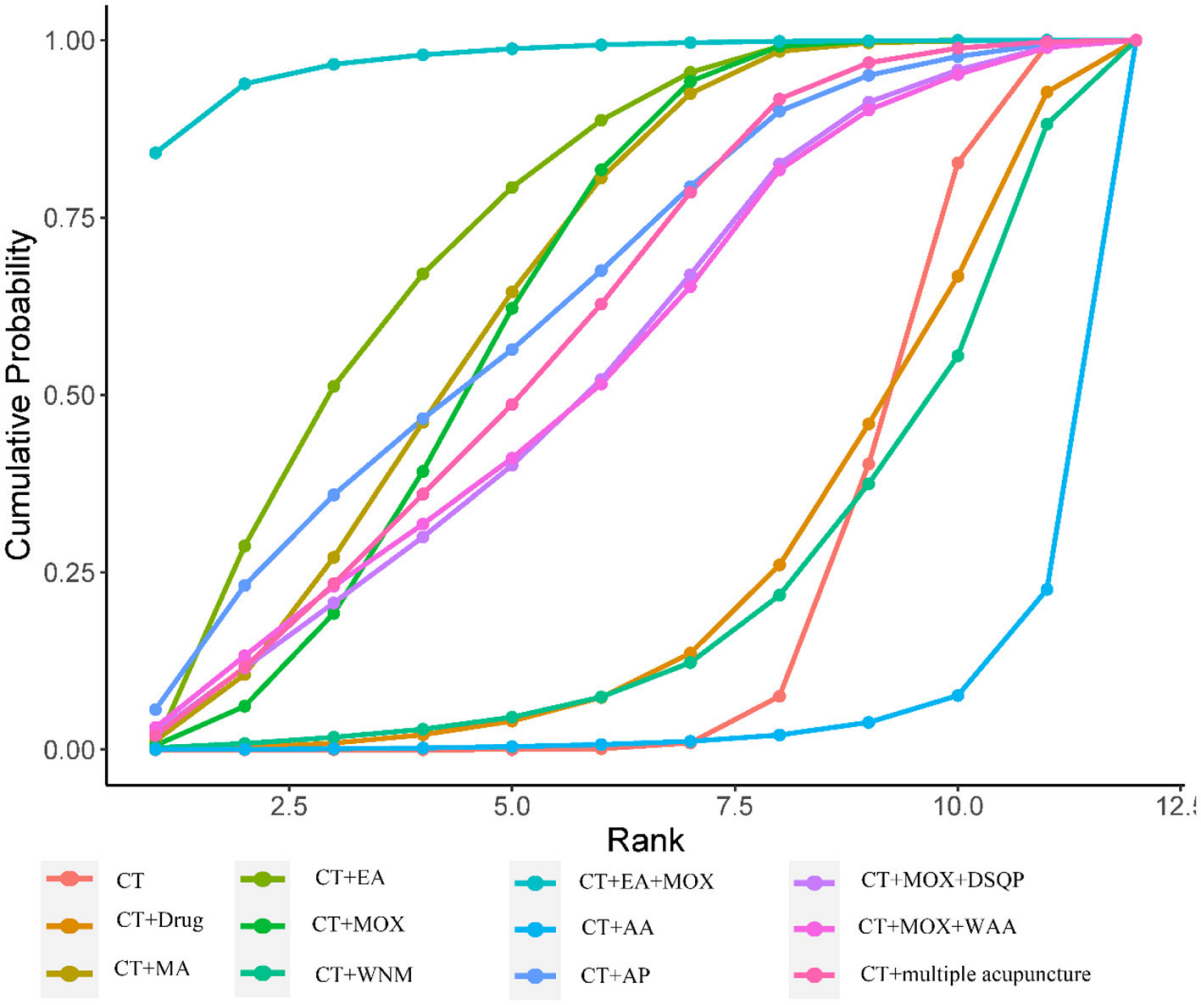
Cumulative probability ranking curve of different interventions for CER. The vertical axis represents cumulative probabilities, while the horizontal axis represents ranks.

Figure 3 shows 12 direct comparisons: CT vs. CT+MA (*n* = 2), CT vs. CT+EA (*n* = 2), CT vs. CT+MOX (*n* = 5), CT vs. CT+AA (*n* = 1), CT vs. CT+AP (*n* = 1), CT vs. CT+DSQP+MOX (*n* = 1), CT vs. CT+WAA (*n* = 1), CT+Drug vs. CT+MA (*n* = 1), CT+Drug vs. CT+EA (*n* = 1), CT+EA vs. CT+EA+MOX (*n* = 2), CT+MOX vs. CT+WNM (*n* = 1), CT vs. CT+multiple acupuncture (*n* = 1). Figure 4 shows the ranked and SUCRA values. CT+EA+MOX ranked first. CT+EA+ MOX (97%) had the highest SUCRA value in CER followed by CT+EA (74%), CT+MA (66%), CT+MOX (64%), CT+AP (63%), CT+multiple acupuncture (59%), CT+WAA+MOX (54%), CT+DSQP+MOX (54%), CT+Drug (24%), CT+WNM (21%), CT (21%), and CT+AA (4%). Table 2 shows the Odds ratio (95%CIs) of all treatments. Compared with CT, CT+MA, CT+EA, CT+MOX, and CT+EA+MOX were associated with significantly higher probabilities of CER.

**Table 2:** League table of all CER comparisons.

A											
1 (0.14, 5.6)	B										
**0.2 (0.05, 0.58)**	0.2 (0.04, 1.11)	C									
**0.15 (0.02, 0.5)**	**0.14 (0.02, 0.73)**	0.73 (0.11, 3.6)	D								
**0.21 (0.08, 0.47)**	0.21 (0.03, 1.49)	1.06 (0.28, 4.1)	1.45 (0.32, 9.05)	E							
1.22 (0.13, 9.74)	1.21 (0.08, 20.55)	6.11 (0.59, 67.35)	8.34 (0.77, 129.5)	5.76 (0.84, 41.94)	F						
**0.03 (0, 0.18)**	**0.03( 0, 0.25)**	0.14 (0.01, 1.22)	**0.19 (0.04, 0.87)**	0.13 (0.01, 1.09)	**0.02 (0, 0.37)**	G					
4.61 (0.68, 35.18)	4.68 (0.37, 82.35)	23.41 (2.7, 271.77)	32.68 (3.62, 515.64)	22.03 (2.92, 228.36)	3.8 (0.24, 77.66)	173.05 (11.93, 4385.33)	H				
0.21 (0.02, 1.47)	0.2 (0.01, 3.46)	1.03 (0.09, 11.74)	1.41 (0.12, 22.08)	0.97 (0.1, 9.35)	0.16 (0.01, 3.39)	7.46 (0.44, 188.98)	**0.04 (0, 0.69)**	I			
0.29 (0.04, 2.06)	0.29 (0.02, 5)	1.47 (0.15, 16.27)	2.02 (0.2, 30.42)	1.37 (0.17, 13.31)	0.24 (0.01, 4.76)	10.43 (0.7, 261.59)	**0.06 (0, 0.98)**	1.46 (0.08, 27.26)	J		
0.29 (0.03, 2.19)	0.29 (0.02, 5.18)	1.48 (0.13, 17.83)	2.04 (0.17, 32.68)	1.39 (0.13, 14.05)	0.24 (0.01, 4.84)	10.65 (0.63, 271.94)	0.06 (0, 1.02)	1.44 (0.07, 28.47)	1 (0.05, 17.38)	K	
0.24 (0.05, 1.05)	0.24 (0.02, 2.79)	1.23 (0.18, 9.09)	1.68 (0.24, 16.9)	1.16 (0.2, 7.01)	0.2 (0.01, 2.78)	8.84 (0.74, 151.26)	**0.05 (0, 0.57)**	1.19 (0.09, 17.04)	0.83 (0.06, 9.88)	0.83 (0.06, 12.16)	L

*A, CT; B, CT+Drug; C, CT+MA; D, CT+EA; E, CT+MOX; F, CT+WNM; G, CT+EA+MOX; H, CT+AA; I, CT+AP; J, CT +MOX+DSQP; K, CT+MOX+WAA; L, CT+multiple acupuncture. The bolded and underlined results indicate statistical significance*.

#### Residual Urine Volume

**Figure 5:**
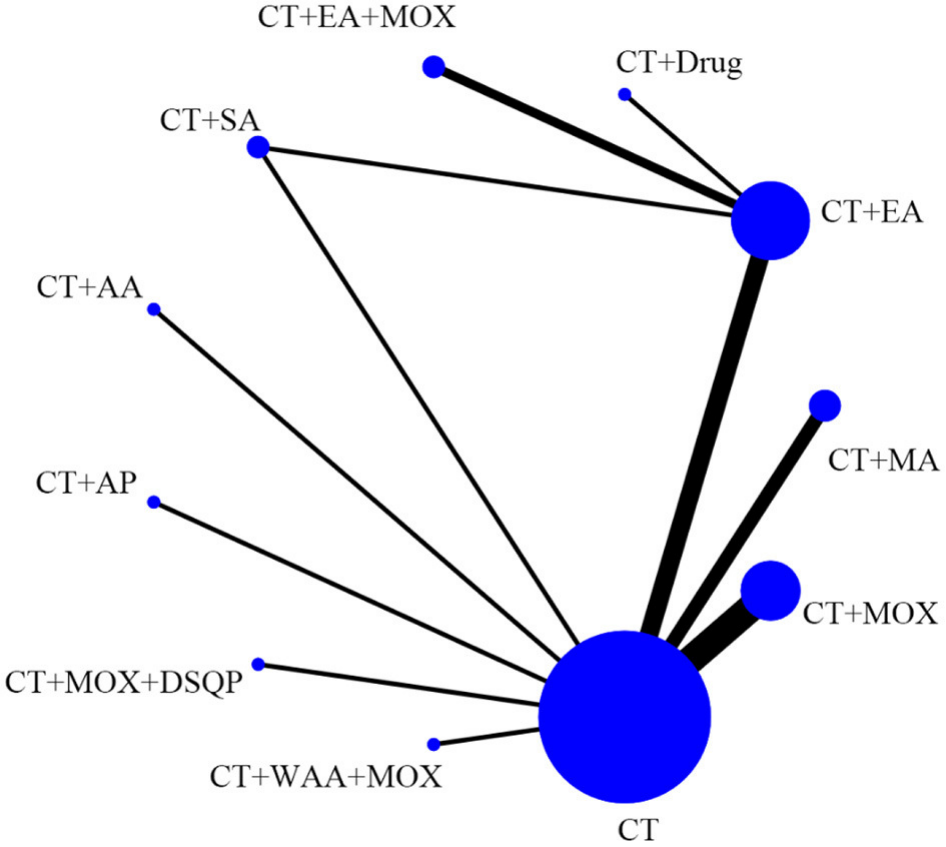
Network of eligible comparisons for the network meta-analysis of RUV. Each node represents an intervention and the size of each node represents the number of randomly assigned participants. Each line represents a direct comparison between interventions and the width of the lines represents the number of studies.

**Figure 6:**
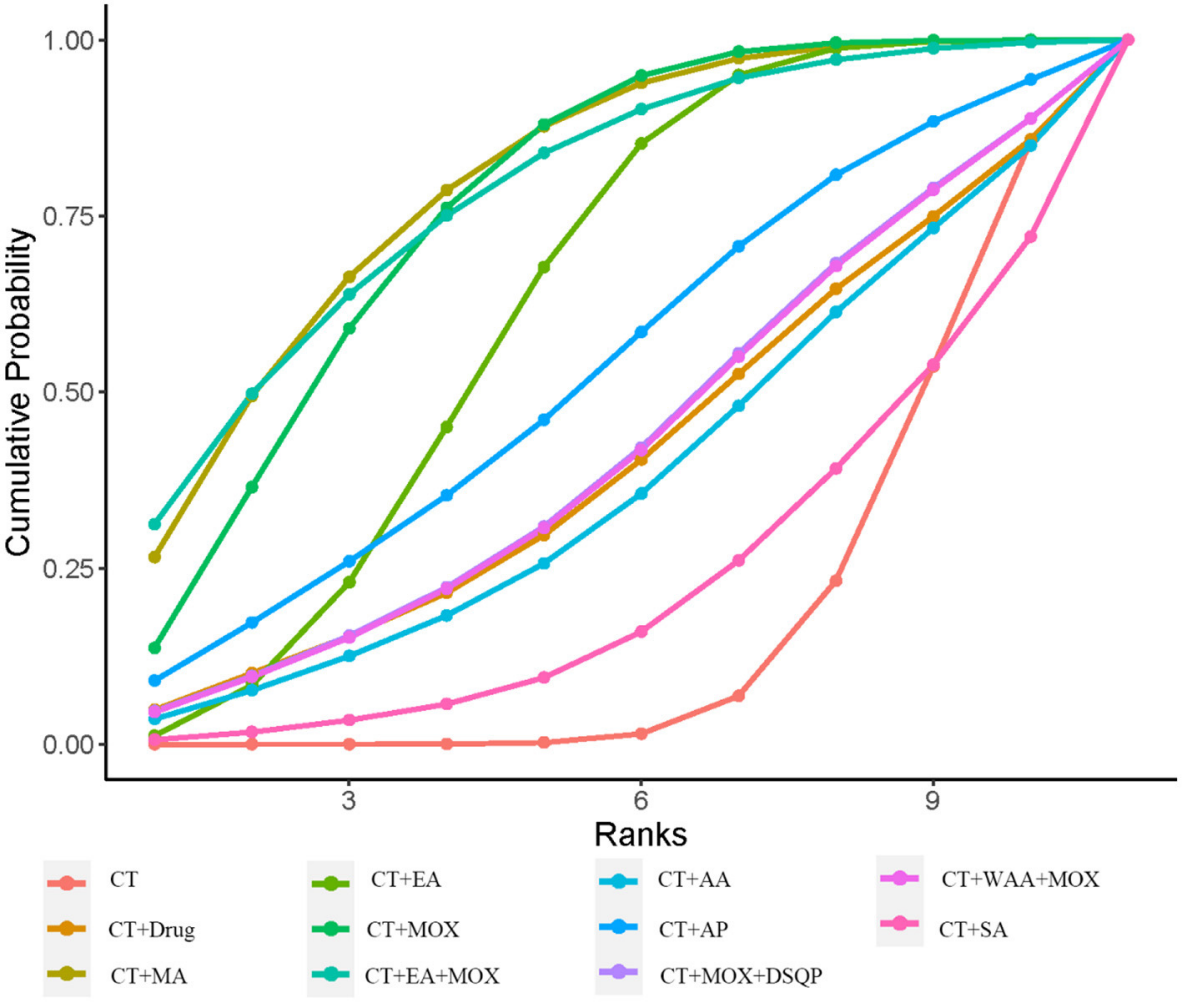
Cumulative probability ranking curve of different interventions for RUV. The vertical axis represents cumulative probabilities, while the horizontal axis represents ranks.

Figure 5 presents 11 direct comparisons: CT vs. CT+MA (*n* = 3), CT vs. CT+EA (*n* = 2), CT vs. CT+MOX (*n* = 6), CT vs. CT+SA (*n* = 1), CT vs. CT+AA (*n* = 1), CT vs. CT+AP (*n* = 1), CT vs. CT+DSQP+MOX (*n* = 1), CT vs. CT+WAA (*n* = 1), CT+Drug vs. CT+EA (*n* = 1), CT+EA vs. CT+EA+MOX (*n* = 2), CT+EA vs. CT+SA (*n* = 1). Figure 6 presents the ranked and SUCRA value. CT+EA+MOX ranked first in terms of RUV and, the SUCRA value followed by CT+MA (79%), CT+EA+MOX (78%), CT+MOX (76%), CT+EA (62%), CT+AP (52%), CT+MOX +DSQP (42%), CT+WAA+MOX (41%), CT+Drug (40%), CT+AA (37%), CT+SA (23%), and CT (17%). Table 3 presents the mean differences (95%CIs) of all therapeutic measures. Compared with CT (control group), CT+MA, CT+EA, CT+MOX, and CT+EA+MOX were associated with significantly higher probabilities of RUV.

**Table 3:** League table of all RUV comparisons.

A										
23.60 (−63.81, 111.61)	B									
**70.12** **(22.72, 119.25)**	46.31 (−53.15, 157.83)	C								
**49.84** **(9.57, 90.42)**	25.91 (−52.84, 104.04)	−20.30 (−84.48, 40.94)	D							
**64.61** **(32.08, 98.67)**	41.03 (-52.07, 135.85)	−5.52 (−64.63, 52.75)	14.99 (−36.88,67.13)	E						
1.78 (−68.05, 72.76)	−21.95 (−126.32, 82.57)	−68.10 (−154.81, 15.96)	−48.11 (−117.91, 23.12)	−62.82 (−141.58, 14.96)	F					
**69.43** **(0.57, 138.31)**	45.55 (−50.50, 141.70)	−0.85 (−85.55, 81.90)	19.65 (−36.04, 74.98)	4.80 (−71.95, 80.83)	67.35 (−21.61, 157.48)	G				
19.32 (−59.55, 99.12)	−4.44 (−122.11, 114.23)	−50.77 (−144.66, 40.76)	−30.58 (−119.57, 59.13)	−45.25 (−131.72, 39.89)	17.46 (−88.45, 123.78)	−49.99 (−153.31, 54.13)	H			
39.10 (−38.70, 117.20)	15.15 (−101.85, 132.81)	−30.67 (−123.61, 58.49)	−10.60 (−98.78, 76.91)	−25.43 (−110.53, 58.72)	37.11 (−66.78, 141.44)	−30.00 (−134.39, 74.46)	19.55 (−89.97, 131.94)	I		
25.14 (−53.74, 103.67)	1.50 (−117.70, 118.38)	−44.86 (−138.94, 44.96)	−24.71 (−113.95, 63.73)	−39.40 (−125.58, 45.18)	23.31 (−82.71, 128.63)	−44.39 (−149.41, 60.15)	6.02 (−107.11, 117.91)	−14.36 (−124.34, 96.35)	J	
24.93 (−53.03, 102.73)	1.05 (−116.61, 118.90)	−45.09 (−137.50, 45.24)	−24.89 (−113.01, 62.16)	−39.67 (−124.92, 43.98)	23.08 (−82.18, 128.34)	−44.54 (−147.88, 59.03)	5.64 (−105.30, 116.23)	−14.03 (−123.79, 96.00)	−0.22 (−11.81, 112.22)	K

*A, CT; B, CT+Drug; C, CT+MA; D, CT+EA; E, CT+MOX; F, CT+SA; G, CT+EA+MOX; H, CT+AA; I, CT+AP; J, CT+MOX+DSQP; K, CT+WAA+MOX. The bolded and underlined results indicate statistical significance*.

#### Publication Bias

**Figure 7:**
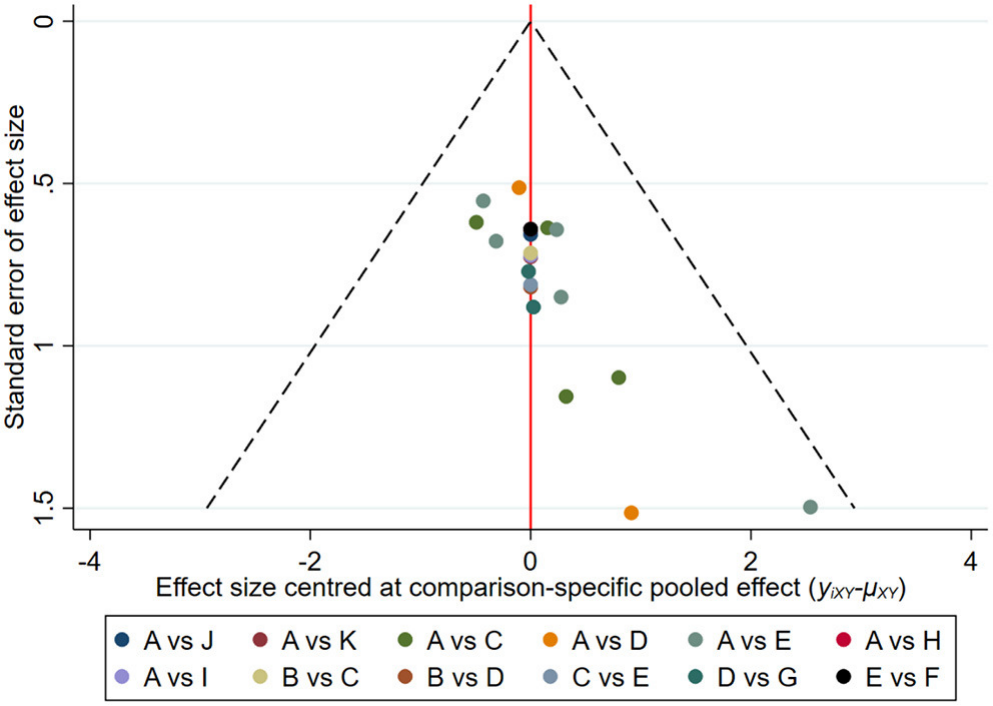
Comparison-adjusted funnel plots for the CER network. The vertical axis represents “standard error of effect size” and the horizontal axis represents “effect size centered at the comparison-specific pooled effect (y_ixy−_ u_xy_).” (A, CT; B, CT+Drug; C, CT+MA; D, CT+EA; E, CT+MOX; F, CT+WNM; G, CT+EA+MOX; H, CT+AA; I, CT+AP; J, CT+MOX+DSQP; K, CT+WAA+ MOX; L, CT+multiple acupuncture).

A comparative adjusted funnel plot was used to assess CER publication bias. When the distribution points in the funnel plot are symmetric, there is no publication bias (52). As shown in Figure 7, all points on the funnel plot were asymmetric and two points were at the bottom of the funnel plot, which represents a potential publication bias.

#### Consistency Test

**Figure 8:**
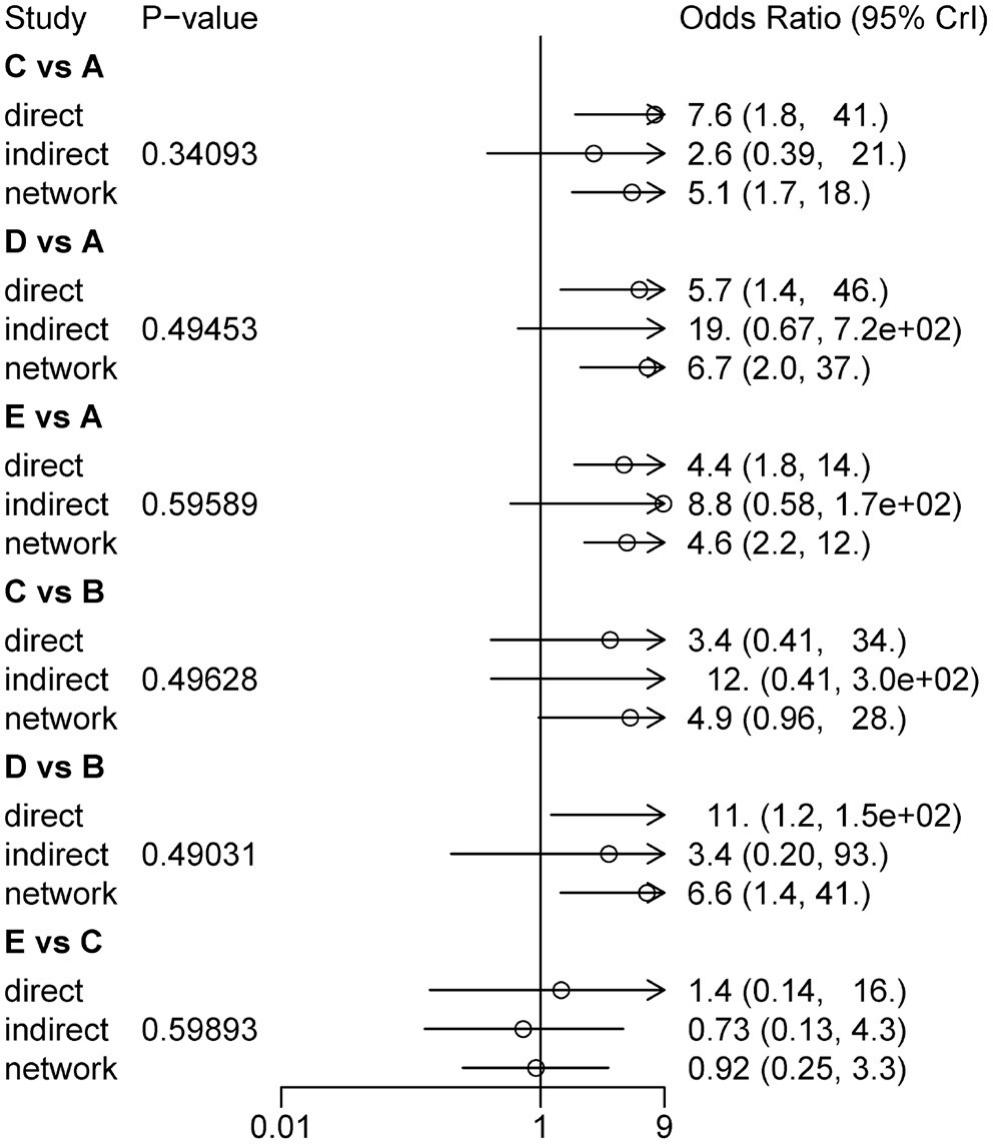
Consistency test results assessed different treatment measures of CER (A, CT; B, CT+ Drug; C, CT+MA; D, CT+EA; E, CT+MOX).

The node-splitting method was used to assess the inconsistency of the model between direct and indirect evidence (53, 54). Closed loops within the network were divided into direct and indirect comparison results. As shown in Figure 8, the results of node splitting revealed that direct and indirect evidence were consistent (*P* > 0.05).

## Discussion

To the best of our knowledge, this is the first Bayesian NMA of acupuncture-related techniques in the treatment of urinary retention in patients with SCI. This study included 26 RCTs. The results of CER in NMA demonstrated that EA combined with MOX, EA, MOX, and MA have significantly increased treatment effects compared with CT. RUV in NMA demonstrated that EA combined with MOX, EA, MOX, and MA have significantly increased positive effects compared with CT. In terms of ranking probability, EA combined with MOX ranked first in both CER and RUV. In terms of cumulative probability, EA combined with MOX ranked first in CER. CER was assessed based on the degree of improvement of TCM clinical symptoms before and after treatment. This evaluation criterion was widely used to evaluate the efficacy of TCM (55–57). Further, the node splitting method showed that the direct and indirect evidence supporting treatment efficacy was consistent. Therefore, EA combined with MOX may be the best acupuncture intervention in patients with urinary retention secondary to an SCI.

The outcomes of the works included in this meta-analysis highlight several important factors, the most important of which is that EA combined with MOX may have better therapeutic efficacy in the treatment of urinary retention due to SCI. There is a good deal of evidence to support this view. An earlier study performed by our team indicated that acupuncture contributed to the recovery of neurologic function after SCI (58), and EA was the most frequently used technique. Another systematic review also suggests that acupuncture was helpful in the treatment of urinary retention after SCI, and EA was also primarily used (15). EA has been found to promote the recovery of bladder function in the setting of multiple pathologies (59, 60). Experimental studies showed that the mechanism of action of EA on bladder function includes apoptosis inhibition, nerve cell protection, and promotion of recovery of injured nerves (61). MOX is also an important part of acupuncture therapy, and has been widely used since ancient times in China. MOX exerts a warm stimulation effect by burning the herb Artemisia vulgaris over an acupoint and is widely considered a type of acupuncture treatment (62). Previous studies have shown that MOX can be used to treat urinary dysfunction caused by a stroke, SCI, or other factor (29, 63–65). Furthermore, EA combined with MOX is commonly used in research and clinical practice (66, 67). It is therefore noteworthy that the cumulative probability of EA combined with MOX did not show a significant advantage over EA alone in terms of RUV. Due to individual variation, RUV may not be the most suitable method for evaluating the clinical efficacy of TCM.

One important factor that needs to be taken into consideration is the selection of a Bayesian method or frequency analysis. The Bayesian method integrates overall information, sample information, and prior information of unknown parameters. According to Bayes' theorem, the posterior distribution of unknown parameters is obtained and unknown parameters are statistically inferred. This flexibility permits the wide use of Bayesian methodology in scientific research. Bayesian NMA also fully considers the uncertainty of parameters and can describe them with direct probabilities (for example, the probability that one intervention is better than another). Compared with frequency methods, Bayesian methods are more valuable when dealing with complex or sparse data (20). Finally, the comparison-adjusted funnel plot was used to detect publication bias in this study. The comparison-adjusted funnel plot appeared to have a degree of asymmetry, suggesting that potential publication bias existed in which “negative” studies were less likely to be reported. Prioritizing the publication of only articles that demonstrated differences between groups might be due to the rejection by journal editors of studies in this research field that report negative findings and seriously limits the quality of the evidence that addresses the effectiveness of acupuncture treatment.

Many other factors are also evident based on the studies we analyzed. First, most studies did not mention the ASIA grade of the SCIs. According to a previous study, ASIA grade on admission affected recovery in both univariate and multivariate analyses (68). We therefore could not explore the homogeneity of the ASIA grade included in these patients. Second, an outstanding question was the course of SCI-mediated urinary retention, as only 20 of our included studies mentioned the specific course of the SCI. Therefore, we were unable to compare differences between the acute and chronic phases. Third, most studies do not report on the long-term efficacy of acupuncture treatments. We therefore could not compare the long-term efficacy of acupuncture treatments. In addition, we could not assess the effect of acupoints on urinary retention due to SCI.

## Limitations

First, the currently available evidence on acupuncture and SCI-related urinary retention is insufficient due to small available sample sizes, limited numbers of patients in each trial, and limited analysis of CER and RUV data. Second, this study did not evaluate the safety of acupuncture because there was a lack of adverse event reporting in most of the included trials. Third, although a comprehensive literature search was performed using multiple online databases, it remains possible that some eligible studies may still have been missed.

## Conclusion

The results of this NWM show that EA combined with MOX may be the most effective acupuncture technique for urinary retention after SCI. Our study may provide an important clinical reference value for clinical investigations of acupuncture in the treatment of neurogenic urinary retention and provide essential information to decision-makers. However, there are too many differences between the designs of the included studies to draw a definitive and clinical recommendation. High quality, large sample size, multicenter clinical trials are needed.

## Data Availability Statement

The original contributions presented in the study are included in the article, further inquiries can be directed to the corresponding author/s.

## Author Contributions

KH and XL were involved in writing and draft preparation. BQ and LJ were involved in literature inclusion and exclusion. RM was involved in writing, draft preparation, and supervision. All authors critically revised the manuscript and approved its final version.

## Funding

This work was supported by the Zhejiang Chinese Medical University Research Fund (Nos. 2019ZG17 and 2018ZY17 to KH); Chinese Medicine Research Program of Zhejiang Province (No. 2019ZZ013 to RM); Zhejiang Provincial Natural Science Foundation of China (No. LQ19H270003 to KH; No. LY19H270009 to RM); National Natural Science Foundation of China (No. 82174487 to RM).

## Conflict of Interest

The authors declare that the research was conducted in the absence of any commercial or financial relationships that could be construed as a potential conflict of interest.

## Publisher's Note

All claims expressed in this article are solely those of the authors and do not necessarily represent those of their affiliated organizations, or those of the publisher, the editors and the reviewers. Any product that may be evaluated in this article, or claim that may be made by its manufacturer, is not guaranteed or endorsed by the publisher.

## Abbreviations

CT: conventional therapy
EA: electro-acupuncture
MA: manual acupuncture
WNM: warm needle moxibustion
AA: auricular acupuncture
AP: acupoint patching
DSQP: dong shi qi point
WAA: wrist-ankle acupuncture
SA: sham acupuncture
SCI: spinal cord injury.
